# Supplementary material for: Efficacy of NAMPT Inhibitors in Pancreatic Cancer After Stratification by MAP17 (PDZK1IP1) Levels
Source: Cancers (Basel). 2025 Aug 5;17(15):2575. doi: 10.3390/cancers17152575 (PMC12346621; doi:10.3390/cancers17152575)
Supplement: Supplementary file 1 [file cancers-17-02575-s001.zip › cancers-3709435-supplementary.pdf]

SUPPLEMENTARY INFORMATION

Supplementary Table S1. Population characteristics and treatment, *n (%)*

	All patients 97 (100%)	Adjuvant therapy Yes 64 (66.0%)	No 33 (34.0%)
**MAP17**			
• 0	40 (41.2%)	-	-
• 1	21 (21.6%)	-	-
• 2	29 (29.9%)	-	-
• 3	7 (7.2%)	-	-
**Age (yr)**			
• Median (range)	67.1 (35-82)	65.1 (36-81)	70.2 (35-82)
• Distribution			
◦ <65yr	39 (40.2%)	29 (45.3%)	10 (30.3%)
◦ ≥ 65 yr	58 (59.8%)	35 (54.7%)	23 (69.7%)
**Gender**			
• Male	55 (56.7%)	35 (54.7%)	20 (60.6%)
• Female	42 (43.3%)	29 (45.3%)	13 (39.4%)
**ECOG**			
• 0	3 (3.1%)	3 (4.7%)	27 (81.8%)
• 1	86 (88.7%)	59 (92.2%)	5 (15.2%)
• 2	7 (7.2%)	2 (3.1%)	1 (3.0%)
• 3	1 (1.0%)	-	-
**Preoperative Ca 19.9 level**			
• Normal	20 (20.6%)	11 (17.2%)	9 (27.3%)
• High	59 (60.8%)	41 (64.1%)	18 (54.5%)
• Unknown	18 (18.6%)	12 (18.8%)	6 (18.2%)
**Pancreatic tumor location**			
• Head	78 (80.4%)	51 (79.6%)	27 (81.8%)
• Body	13 (13.4%)	9 (14.1%)	4 (12.1%)
• Tail	6 (6.2%)	4 (6.3%)	2 (6.1%)
**Surgery**			
• Whipple resection	63 (64.9%)	42 (65.6%)	21 (63.6%)
• Total pancreatectomy	15 (15.5%)	8 (12.5%)	7 (21.2%)
• Distal pancreatectomy	19 (19.6%)	14 (21.9%)	5 (15.2%)
**Histology**			
• Adenocarcinoma	97 (100.0%)	64 (100.0%)	33 (100.0%)
**Tumor grade**			
• Well-differentiated	22 (22.7%)	12 (18.8%)	10 (30.3%)
• Moderately differentiated	61 (62.9%)	41 (64.1%)	20 (60.6%)
• Poorly differentiated	10 (10.3%)	9 (14.1%)	1 (3.0%)
• Unknown	4 (4.1%)	2 (3.1%)	2 (6.1%)
**T**			
• pT1	5 (5.2%)	2 (3.1%)	3 (9.1%)
• pT2	14 (14.4%)	10 (15.6%)	4 (12.1%)
• pT3	65 (67.0%)	45 (70.3%)	20 (60.6%)
• pT4	11 (11.3%)	7 (10.9%)	4 (12.1%)
• Unknown	2 (2.1%)	-	2 (6.1%)
**N**			
• N0	37 (38.1%)	22 (34.4%)	15 (45.5%)
• N+	59 (60.8%)	42 (65.6%)	17 (51.5%)
• Unknown	1 (1.0%)	-	1 (3.0%)
**Tumor stage**			
• I	10 (10.3%)	5 (7.8%)	5 (15.2%)
• II	73 (75.3%)	50 (78.1%)	23 (69.7%)
• III	14 (14.4%)	9 (14.1%)	5 (15.2%)
**Adjuvant therapy**			
• Adjuvant CT	52 (53.6%)		
• Adjuvant CTRT	11 (11.3%)		
• Adjuvant RT	1 (1.0)		
• No	33 (34.0%)		
**Follow-up (mo)**			
• Median (range)	20.4 (6.7-166.2)	23.6 (8.0-166.2)	15.9 (6.7-124.9)
**Relapsed**			
• Yes	78 (80.4%)	50 (78.1%)	28 (84.8%)
• No	19 (19.6%)	14 (21.9%)	5 (15.2%)

Ca 19.9: carbohydrate antigen 19.9. CT: Chemotherapy. RT: Radiotherapy.
+3 are +2 by IHQ; +2 are from 1.01 to 1.99; +1 are from 0.5 to 0.99; 0 are from 0 to .49 + lost

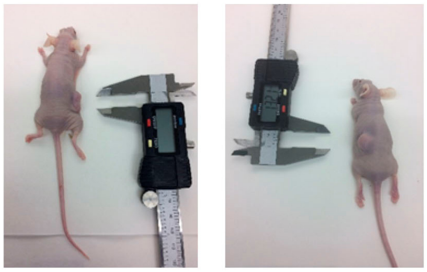

Supplementary Figure S1. Representative images of tumor formation.

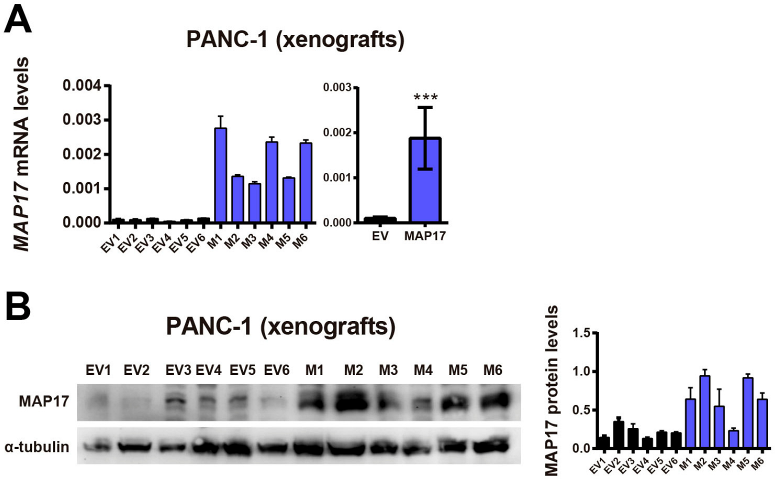

Supplementary Figure S2. Validation of the overexpression of MAP17 in tumors derived from PANC-1 xenografts. A) Measurement of MAP17 expression by RT–qPCR in all the tumors derived from PANC-1 xenografts. We represent the individual expression of all the tumors (left) and the mean of the expression of each group (right). **B)** Measurement of MAP17 protein levels by western blot. Representative images of western blot analysis are shown (left). Protein levels were quantified and normalized according to α-tubulin levels (right).

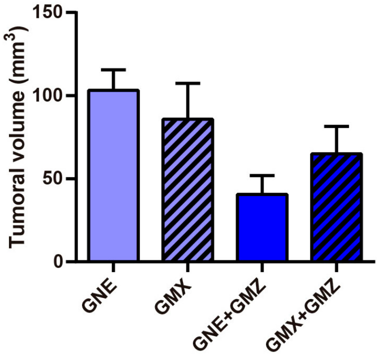

Supplementary Figure S3. Comparison of the *in vivo* effect of the two inhibitors of NAMPT in pancreatic tumors. Determination of the tumor volume in xenografts derived from PANC-1 cells that overexpressed MAP17 (N=4) after treatment with GMX1778 or GNE617 alone and in combination with gemcitabine (GMZ). Figures show the statistical comparisons between groups in the end point (final tumor volume) of Figure 6.

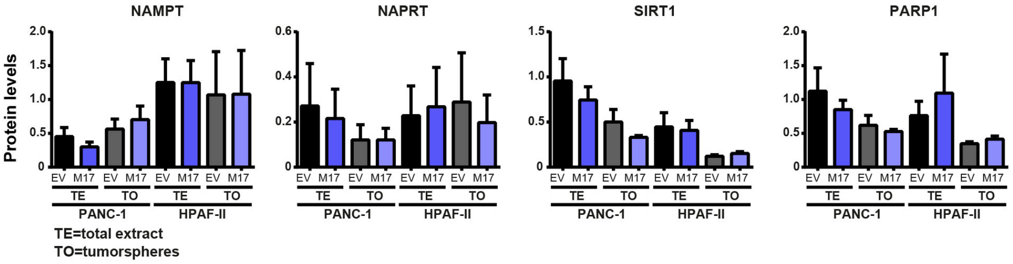

Supplementary Figure S4. A) Measurement of the protein levels of NAMPT, NAPRT, SIRT1, PARP1 and HSP70, which was the loading control, in the total extracts and tumorspheres derived from PANC-1 and HPAF-II control and MAP17-overexpressing cell lines by western blot analysis.

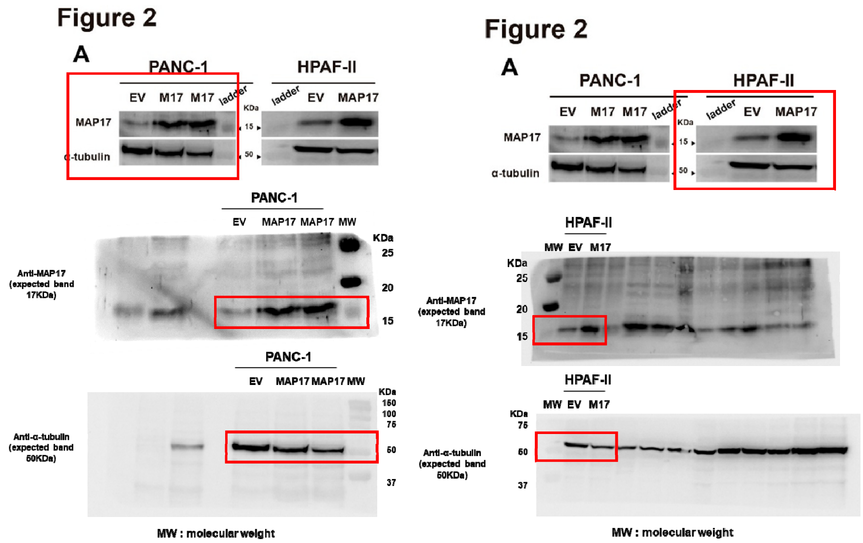

Intensity ratio						
**PANC-1**	**EV**	**MAP17**	**MAP17**	**HPAF-II**	**EV**	**MAP17**
**MAP17**	3196949	8166444	8724170	**MAP17**	4256591	12098080
**tubulin**	20224288	12410970	7238624	**tubulin**	7817744	5130560

Supplementary Figure S5: Original Western bolt figures and intensity ratios.

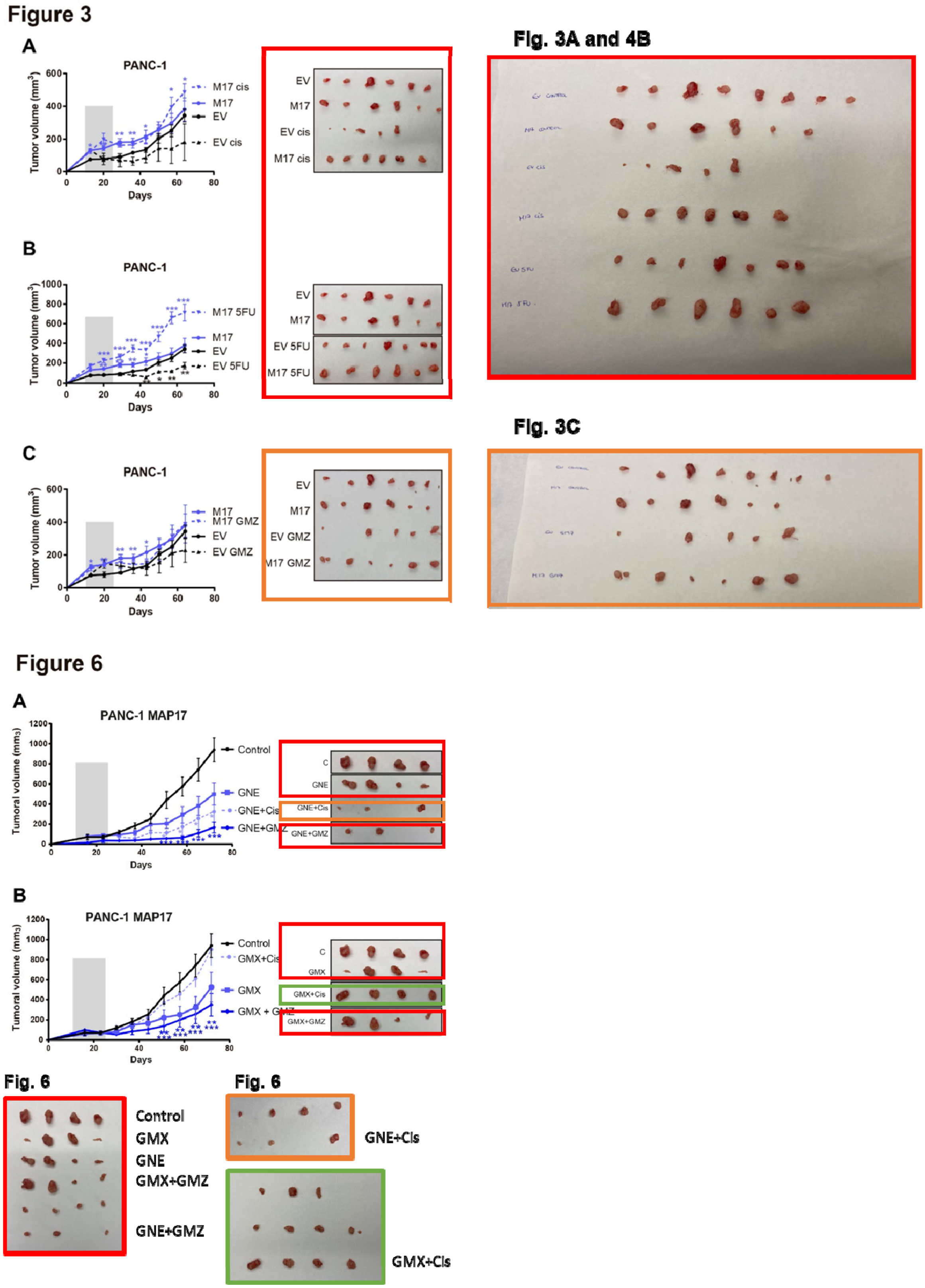

Supplementary Figure S6: Original *in vivo* images
